# The success elements of humor use in workplace leadership: A proposed framework with cognitive and emotional competencies

**DOI:** 10.1371/journal.pone.0304650

**Published:** 2024-05-31

**Authors:** Caroline Rosenberg, Catherine L. Caballero, Alexa Hayley, Arlene Walker

**Affiliations:** Workplace Wellbeing Innovation and Performance Group, School of Psychology, Department of Health, Deakin University, Geelong, VIC, Australia; Universita degli Studi di Perugia, ITALY

## Abstract

This qualitative study aims to investigate the competencies and effectiveness of humor use in workplace leadership. By exploring the elements underlying successful and unsuccessful humor use, this research offers insights into the competencies required for leaders to leverage humor effectively. Adopting a qualitative inductive approach, fifteen individual semi-structured interviews were conducted, generating a dataset of 51 critical incidents of humor use. Reflexive thematic analysis was employed to identify key themes, resulting in the identification of five central elements: Reading the context, Intention and motivation, Judgement and decision, Skillful delivery, and Understanding reactions. These elements provide a comprehensive framework for understanding humor use in the context of workplace leadership, emphasizing the importance of cognitive and emotional intelligence / competencies. The study proposes a theoretical framework based on these findings, providing the foundation of a new paradigm for understanding and measuring humor use. This research contributes to a deeper understanding of the competencies and complexities involved in using humor as a leadership tool and provides practical implications for leaders aiming to enhance their leadership effectiveness through humor.

## 1 Introduction

*Humor is the great thing*, *the saving thing after all*. *The minute it crops up*, *all our hardnesses yield*, *all our irritations*, *and resentments flit away*, *and a sunny spirit takes their place*.

                •Mark Twain

*Humor must not professedly teach, and it must not professedly preach, but it must do both if it would live forever*.

                •Mark Twain

Interest is growing in understanding the use of humor in workplace leadership, with encouraging evidence of its positive influence on leadership effectiveness and workplace outcomes [1, 2]. A scoping review conducted by Rosenberg et al. (2021) examined the evolution of research on humor in workplace leadership over the past four decades. Four broad categories of research were identified, encompassing the relationships between humor and workplace outcomes, qualitative investigations of humor as a communication tool, moderators and mediators of the humor-leadership relationship, and the influence of culture on humor [3]. This complexity highlights the need to further explore humor use as a critical leadership skill and its underlying competencies.

Quantitative research in this domain has predominantly relied on styles-based measures, with Martin et al. (2003)’s Humor Styles Questionnaire (HSQ) being the most commonly used instrument in humor assessment. However, the HSQ framework was primarily developed to investigate humor from an individual wellbeing perspective, and so its utility in a leadership context may be limited. Leaders may recognize the need to adapt their humor style, but existing frameworks do not provide insight into style adaptation or the necessary skills for using humor more effectively.

A meta-analysis by Kong et al. (2019) found that leaders’ expressed humor behaviors have a stronger impact on follower outcomes, such as positive emotions and trust, compared to their trait humor. This suggests that humor use can be developed, that is, leaders who learn how to express humor effectively can harness its positive effects, even if they are not naturally inclined towards using humor. This aligns with the social phenomenon perspective of humor, whereby humor is created by society, constructed by people, and involves observable behavior used to influence others.

The theoretical background for humor studies has evolved over the decades. A detailed description can be found in the scoping review by Rosenberg, Walker [3]. Humor theories can be summarized into four categories: cognitive, emotional, social and workplace related. Cognitive based theories cover both the causes and functional purposes of humor (e.g., relief theory). Emotional based theories mainly focus on the consequences of humor (e.g., emotional contagion theory). Social interaction theories position humor as a key individual resource that contributes to interpersonal functions (e.g., social exchange theory). In workplace related theories, humor is conceptualized as a resource for coping with job demands and facilitating relationships (e.g., Leader-Member Exchange theory).

In addition to above mentioned, there have been two theoretical models proposed that were directly related to humor in the workplace. The Relational Process Model by Cooper (2008) explains how humor operates to affect relationships in the workplace. The Wheel Model of Humor by Robert and Wilbanks [4] suggests that humor perpetuates a positive affect on individuals and groups, and creates a climate that supports subsequent humor events. While these models may be valuable to leaders in highlighting the positive impact of humor use in the workplace, they do not elucidate the leadership skills and competencies required to create and harness a positive effect using humor. Therefore, it is imperative to develop humor theories or frameworks specific to the needs of effective leadership in the workplace.

Despite the current theoretical gap, the empirical studies on humor have demonstrated the significant value humor brings in creating positive workplace outcomes at various levels of analysis. At individual level, humor alleviates stress [5] and promotes wellbeing and innovative behavior [6]. As humor is closely connected with laughter, humor is also recognized for its biochemical benefits, such as reducing cortisol levels [7]. At relationship level, humor reduces social and power distance [8] and facilitates trust development [9]. At an organisational level, humor contributes to enhanced ability to manage organisational change [10], and improved employee psychological capital and wellbeing [11]. There are several review studies that documented the impact of humor in detail, such as Holmes and Marra [12], Rosenberg, Walker [3]. This evidence of positive humor impact further emphasizes the need to understand how humor can be developed, used effectively, and the need to identify the key elements of humor use that lead to positive versus negative outcomes.

The contrast between the playfulness of humor and the seriousness of work is encapsulated by the phrase “humor is a double-edged sword”, as it highlights the duality in humor functions between unification and division [13–15]. Studies show humor production ability is related to verbal intelligence [16, 17], and the use of humor signals confidence, however, the inappropriate use of humor signals incompetence [8]. There is recent evidence to suggest humor use only affects emotional connection (warmth) rather than judgement on competence [18]. Despite the inconsistent findings, these studies suggest effective humor use may involve both cognitive and emotional intelligence.

The purpose of this study was to explore humor use in a workplace leadership context to broadly understand the key elements that influence successful humor use, in other words, the elements of humor use that may lead to positive outcomes. Also, how these elements can influence perceptions of both leaders and followers regarding effective leadership. The study had three objectives. First, to identify key elements important for perceived success of humor use in a workplace leadership context. Second, to postulate how success elements underpinning humor use relate to or influence leadership effectiveness. Third, to use the findings to inform the development of a framework for effective humor use in a workplace leadership context. By addressing these objectives, this study intends to contribute to the existing literature by advancing the understanding of humor use in the context of workplace leadership and providing practical insights for leaders to enhance their leadership effectiveness through humor.

## 2 Method

This study used qualitative inductive methodologies to explore and develop new understandings of humor without the restrictions of known definitions and measures. There were two key considerations in designing this study. First, fundamentally influenced by Heidegger’s phenomenology [19], it was important to look beyond the simple “funniness” of humor in leadership and unveil the reasons for or ‘meaning of’ humor use from individual experiences. This study adopted a critical realist ontology–humor use can be constructed via thought and culture within a material world that exists independently. The study was designed with an empirical epistemology and an interpretative-constructive lens–humor use can be studied directly and indirectly via examining observations and logic, however, subjective interpretation of humor use had to be used to construct its meaning. This combination of ontological and epistemological perspectives was suitable for the subjective interpretation of the empirical data, while drawing on past scientific knowledge to interpret and extend its meaning. The second consideration was to ensure information about humor use could be consistently gleaned from participants as a means of generating theory about humor use. Critical incident technique (CIT; Byrne, 2001) was used as part of the interview process to elicit consistent information from participants about humor use experiences and reasons for use, and is explained in greater detail below.

### 2.1 Participants

There is no single best solution in determining sample size for qualitative research, because it is driven by the purpose of the research, quality of the data collected, and sampling strategy used [20–22]. However, data saturation is one principle commonly used to determine sample size in qualitative research [20]. Hennink, Kaiser [23] found that in-depth interviews reach code saturation (based on descriptions) at nine interviews; however, meaning saturation (based on interpretations) were reached at 16 to 24 interviews. Terry and Hayfield [24] also suggested that six to ten participants might be sufficient for interview studies where each participant is likely to make a considerable contribution, resulting in a rich dataset. In the present study, the aim was to recruit approximately 20 participants. However, no new codes or themes were identified from the data at 15 interviews, indicating data saturation was achieved. Consequently, data collection was ceased, as the sample size was deemed sufficient to address the research objectives and generate a comprehensive dataset.

Participants (*n* = 15) completed in-depth interviews [25, 26], ranging between 45 and 90 minutes. Participants were aged between 25 and 60 years, of which 10 fell within the 35–45 year age group. Six of the participants were female and nine participants were male, representing sectors such as manufacturing, construction, consulting, government, and not-for-profit organizations. Participants worked from a range of locations in two States in Australia (Victoria and South Australia). Thirteen participants were working in leadership positions, and two were team members who reported observations of their leaders’ humor use behavior.

### 2.2 Procedure and data collection

The recruitment period for this study commenced on January 9^th^, 2019, and concluded in October 2019, as the analysis of the 15^th^ interview transcript indicated data saturation. A snowball sampling method was used to recruit participants through the researchers’ personal and professional network. The aim was to recruit participants who were working in an Australian organization with experience in leadership positions–a role that has at least one direct-repot. However, participants who were not in a formal leadership position were not actively excluded from the study, instead, these participants were asked to provide insight from a follower/observer perspective.

Participants were purposively recruited from different industry sectors to minimize selection bias and achieve greater heterogeneity of data sources. Three interviews were conducted over the phone, and the other twelve were conducted in person. The interviews were audio-recorded and later transcribed verbatim by the researcher. Identifying information was removed from transcripts to ensure anonymity of the participants.

#### 2.2.1 Semi-structured interview

A semi-structured interview schedule was used, which allowed consistent exploration of research questions with participants, as well as flexibility to engage with the phenomenon differently based on the unique responses of each participant [27]. The interview questions were designed to elicit introspective information, individual experiences relating to personal and other leaders’ humor use, individual reflections about specific humor incidents, and elements that contributed to personal perceptions and experiences (See S1 Appendix).

At the outset of the interview, participants were asked to self-identify into one of two groups: leader or follower. This allowed the participant to self-select into the follower group if they did not self-identify as a leader, even if they had experience in a leadership position. The same semi-structured interview schedule was used for both groups, with the leader group (*n* = 13) asked to reflect on personal leadership and humor use experiences as well as observations of other leaders’ humor use.

#### 2.2.2 Critical Incident Technique

Critical Incident Technique (CIT) was used to elicit real life examples from the participants that could contribute to understanding how leaders and followers experience humor use in the workplace. CIT is a method of collecting descriptions (both positive and negative) of events or occurrences (termed “incidents”) (Byrne, 2001; Radford, 2006). The described incidents are analyzed to gather information about how they influence a particular outcome [28]. According to Flanagan (1954), a described critical incident should contain context, behaviors, and outcomes. Use of CIT in the current study allowed for separate exploration of the elements involved in successful and unsuccessful humor by: 1) identifying the circumstances of using humor in a leadership situation (context), 2) what helped or hindered the use of humor in the situation (behaviors) and, 3) what the outcomes were (positive or negative) for the leader or followers (outcomes). The researcher was able to compare and contrast the described incidents of humor use to identify common themes and patterns relating to using humor in a leadership context. This methodology allowed for a nuanced exploration of the elements involved in humor use, providing valuable insights into the dynamics of workplace humor and its impact on leadership effectiveness.

### 2.3 Data analysis strategy

**Fig 1:**
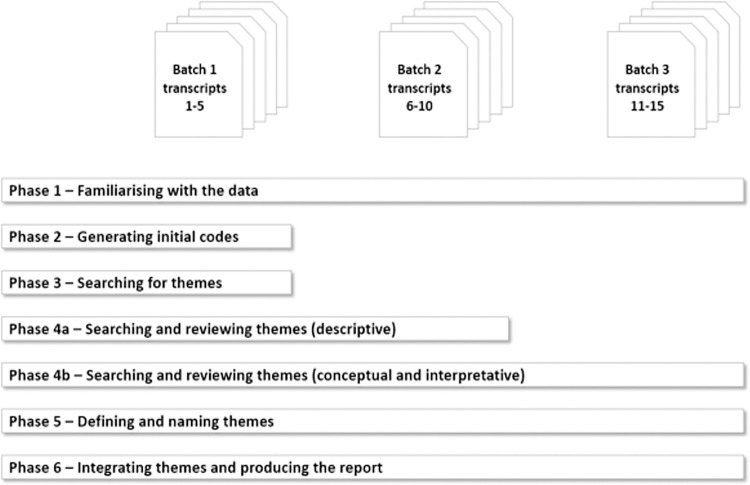
The adapted six-step thematic analysis process.

A reflexive thematic analysis approach was used to analyze the dataset. This was adapted to incorporate reflexive identification of theories–the aim of this was to generate conceptual themes that required the researcher to interpret meaning from the text [29, 30]. The 255-page text file of transcripts was analyzed following the 6 steps recommended by Braun and Clarke (2006) illustrated in Fig 1. In the first step of the thematic analysis process, following transcription of the interviews, the researcher reviewed the 15 transcripts. To manage the breadth of data, and maintain consistency and depth of analysis, the researcher chronologically divided the transcripts into three batches based on the interview dates and coded the transcripts. The unit of analysis was clusters of complete sentences, where one complete idea was described [31]. There might be multiple ideas expressed within the response to a specific question asked, and there might be multiple sentences involved in articulating that idea. Coder discussion and semantic agreement was used to determine trustworthiness of coder interpretation. Steps 2 and 3 of the analysis process used the first batch of transcripts (1–5) to inductively generate initial codes and themes. Step four of the process was split into two parts. The first part focused on reviewing the descriptive themes generated across the first two batches of transcripts (1–5 and 6–10) and the second part involved searching and reviewing the descriptive and interpretative themes across all three batches of transcripts (1–5, 6–10 and 11–15). Data saturation was reached at this stage. Step five involved defining and naming the themes. A clear description of each theme was defined in this step. Finally, the themes were integrated into a coherent framework in step six.

In addition to the steps outlined above for data analysis, additional steps were taken to ensure the process of the qualitative analysis was rigorous and trustworthy [32]. To demonstrate credibility and dependability of the data analysis, another researcher, who was not familiar with the study, double coded 80% of the dataset deductively using the definitions and inclusion criteria provided in the codebook created for this study. Both sets of coding provided traceable and clearly documented evidence for the trustworthiness of the analysis. The purpose of the double coding process was to provide clarity around definitions of themes rather than reaching a consensus about coding [33]. Therefore, disagreement in coding were reviewed, discussed collaboratively, and differences were resolved by revising the definitions or labels of the themes.

### 2.4 Ethics statement

The Deakin University Health Ethics Advisory Group granted approval for this study with project reference HEAG-H_175_2018. All participants were provided a plain language statement about this study and a written consent form to sign prior to the interview. All participants were informed that they may withdraw from the study at any time without consequence. This study did not involve any minors.

## 3 Results

**Fig 2:**
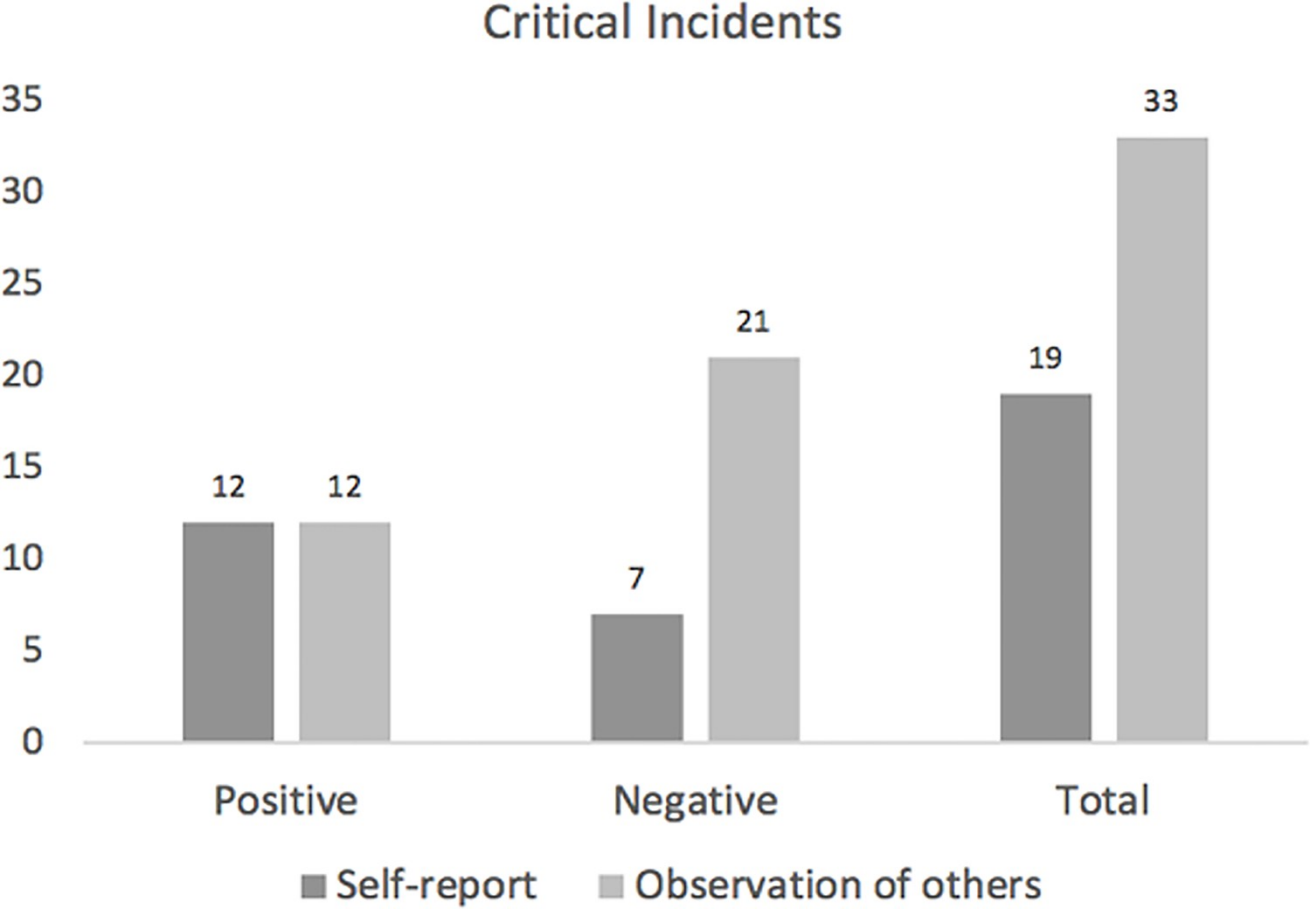
The number of critical incidents reported for humor use in the workplace leadership context, breakdown by perceived positive or negative outcome and comparisons of self-report versus observational incidents.

The qualitative analysis of the 15 interviews resulted in the generation of a total of 51 critical incidents related to humor use in workplace leadership contexts. These critical incidents included both successful use of humor–incidents that led to positive outcomes; and unsuccessful use of humor–incidents that led to negative outcomes. Among the critical incidents, an equal number of positive humor use incidents were reported based on personal experiences and observations of others, with 12 incidents each in these categories. However, more examples were provided of negative humor use incidents when it came to observations of other people, with 21 incidents reported. In contrast, self-report descriptions showed an underrepresentation of negative humor use incidents, with only 7 incidents reported. Overall, there were over 80% more observational than self-report incidents of humor use in the workplace leadership context. (see Fig 2).

The themes and subthemes identified from the Reflexive Thematic Analysis are summarized and presented in Table 1. Example quotes from the interview transcripts are used to illustrate the subthemes in the written descriptions that follow. Each quote is tagged as “self-report” or “observation”, and because leaders could also report observational incidents of other leaders, “leader” and “follower” are also used to further differentiate the role of participants in each described incident. For example, if a leader described an incident of humor use that they observed from another leader, the quote is tagged “Leader observation”.

**Table 1:** Themes and sub-themes of humor use in the workplace leadership context.

Themes	Sub-themes
Reading the context	The people (including relationship)
	The task (seriousness)
	Context type (open or closed context)
Intention and Motivation	Clear purpose of humor use
	Awareness of motivation
Judgment and Decision	Principles of judgement
	Speed of judging and decision-making
Skillful delivery	Spontaneity/rehearsal
	Communication style
Understanding reactions	Understanding negative reactions
	Understanding perceptions and inferences

Five themes were identified as pivotal to successful humor use: *reading the context*, *intention and motivation*, *judgement and decision*, *skillful delivery*, and *understanding reactions*. Descriptions are provided below to illustrate the themes and sub-themes.

### 3.1 Reading the context

The ability to read the context emerged as a common element in successful humor use. There were three sub-themes related to “the context”: *the people*, *the task*, and *context types*. The first sub-theme was about understanding the people in the audience.

*There’s a lot of arrows in a humor quiver*, *there’s self-deprecation*, *there’s just making fun of somebody*, *there is all these different areas that you can use*, *you have to read and understand the person before you start using it*. [Leader Observation]

Understanding the people also included understanding the nature of the relationship between the leader and the people involved in the situation.

*They were the clients*, *we’d only met them for about 30 seconds*, *and he dropped the joke that*… *it wasn’t even funny*. [Follower Observation]

The second sub-theme was about the task at hand, the purpose of the interaction, and the objectives of the other person. In certain situations, some participants noted that humor was not the best option:

*So*, *they were trying to elicit information from me… humor could detract the effectiveness of the conversation …because ultimately*, *you’re just trying to convey some sort of message*, *whether you use humor or not*, *you want to find the most effective way to get that through*. *…by reading the other person*, *you can try to work through your toolbox of ways of approaching it*. [Leader Self-report]

The seriousness of the task at hand may also determine the appropriateness of humor use in that situation.

*I think no one laughed because I probably read the room wrong because it was quite a serious conversation and probably people weren’t expecting it to be funny*. [Leader Self-report]

The third sub-theme was related to the context type, open or closed. Open contexts were public, often perceived as high risk; whereas closed contexts were private, away from others, and perceived to be safe and controllable. People appeared to consciously choose different types or styles of humor to use in public versus private.

*In a public forum*, *he would make a couple of jokes*, *but it wasn’t ’till behind closed doors*, *when it was one on one*, *or one on two*, *did you really appreciate the type of humor that he uses*. *He wouldn’t come to the pointy end of the humor… making a joke about another colleague or something like that until [behind] closed doors*. [Leader Observation]

On the other hand, the open and closed contexts could change how a person felt and reacted to the same humor used by the same leader, because in private, the person may feel safe to be the butt of a joke, and react positively; however, when the same joke was used in public, the person may feel embarrassed and in turn react negatively.

*I had said a joke and I meant it [to be] funny*, *but it hurt them… because it was publicly done*, *in a room*, *they said "but people looked at you and thought you were serious*.*”* [Leader Self-report]

### 3.2 Intention and motivation

The second theme that emerged in successful humor use was the intention and motivation for using the humor. There were two sub-themes identified: the *clear purpose of humor use* and *awareness of motivation*.

For some, the purpose of using humor was clear, to assist in building positive relationships with others, as illustrated below.

*It’s actually easier to make people deal with difficult things if they like you*, *and it’s very difficult to not like somebody who you’re laughing with*. *If they don’t know you*, *so if they don’t know if they like you*, *yet*, *but you make them laugh*, *I think they’re more likely to like you*, *then can lead to trust*, *can lead to whole lot of other things*. [Leader Self-report]

However, even when the intention of using humor was clear, self-awareness of the intrinsic versus extrinsic *motivations* appeared to influence if or how humor was used. For example:

*[When using humor] do I come from a naturally heart felt sort of compassionate caring position*, *or do I come from real head based [position]… I’m gonna say a joke and if people find it offensive*, *then so be it*. *It’s different [motivation]*. [Leader Self-report]

Clear intention may not always lead to a positive outcome for humor use; however, when a negative outcome occurs, the causes of the negative outcome are likely to be misreading the cues and information gathered through *reading the context*, or the situation has changed.

*Most people do not actually want to cause offence*, *even people who might say something inappropriate…don’t want to cause offence*, *they got caught out because somebody who wasn’t supposed to hear it*, *heard it*. [Leader Observation]

If the leader is not self-aware of their motivations and objectives for using humor, or the intention of using the humor is not clear to the audience, it can lead to confusion and uncertainty.

*I don’t know when he’s joking when he’s not; when he’s really joking and when he is serious about stuff*. [Follower Observation]

For some, when the intended purpose of humor was not clear, it was dismissed as wasting time.

*I think sometimes he’s just trying to be funny but [I] didn’t really get the impact at all*. *It’s just a waste of time for me to listen…I could have done more productive stuff rather than listening to the jokes*. [Follower Observation]

### 3.3 Judgment and decision

The third theme identified relating to successful humor use in a workplace leadership context was *judgment and decision*. Participants reported that the principle used for judgement in decision-making about using humor was important. The following example illustrates keeping people safe as an important principle of judgement.

*This comes back to the whole point of how I keep myself and others safe*. *If I was going to crack a joke about your gender*, *because I was in a room full of blokes*, *and I thought they’re all gonna find that funny*, *then that’s not an appropriate use of humor*. [Leader Self-report]

Some leaders noted that, despite awareness of the situation and people involved, they often lacked speed in judging appropriate humor use in a situation. For example:

*I know that person is sensitive*, *but again… whatever is going through my brain was filtered out*, *and I didn’t use my judgement to stop myself in time*. [Leader Self-report]

The decision-making process appeared to include judging how much humor to use, evaluating the relationship quality against the nature of the situation, and anticipating outcomes.

*I would actually almost say*, *as soon as you think it’s something people expect from you*, *you’re probably doing it too much*… *and it’s probably become less useful*. [Leader Observation]

*I suppose there’s a risk if you’re always trying to be best friends*, *play games with your employees and staff*, *sometimes*, *it might actually backfire a little bit*. [Observation as follower]

### 3.4 Skillful delivery

Participants noted leader skill in delivering humor as being useful. Skillful delivery was described by participants in different ways, including voice-based skills, such as intonation, and body language, such as gestures and facial expressions. For example,

*You gotta know when to pause*, *when to look sidewards*… [Leader Self-report]

Participants also reported humor as a way of communication. Fundamental communication skills were found to be applicable to humor delivery. For example, presenting with confidence.

*If you know the individual… is not too confident… ’coz humor obviously involves everyone paying attention to you*, *being the center of attention*, *if you don’t like that*, *you’re not gonna want to say the zinger*… [Leader Observation]

Skillful humor delivery was also encapsulated in the concept of spontaneity.

*It’s always unexpected*, *in the sense that you know*, *he’s just able to think quick on his feet and say something that is applicable to the situation and yet eases tension*. [Follower Observation]

### 3.5 Understanding reactions

The final theme related to understanding reactions to humor use, in particular negative reactions, and how well the leader understood and responded to these reactions.

*She thought it was hilarious*, *but that rest of the room was silent [was lost on her]*, *and it was awkward… There was the silence*, *and then there was*, *the facilitator… jumping in and trying to quickly divert the attention and change subjects*. [Leader Observation]

A common negative reaction involved silence or perceived awkwardness, as illustrated in the following example.

*He made a joke*, *it was a sexist joke about a woman secretary… they were silent*, *they tried to move past it very quickly*, *but the temperature of the room changed completely*, *from "nice to meet you" to just no volume… and from then on in*, *it was always awkward*. [Follower Observation]

How the leader reacted to unexpected or negative outcomes of their humor use was deemed to reflect their personal characteristics.

*"So*, *you know I have had people visibly offended [by my humor] and I will straight away say ’I’m so sorry*… *that came out wrong’*… *Sometimes If I try and be funny and no one laughs I just move on*, *no harm no foul*.*"* [Leader Self-report]

Sometimes, perceptions of negative outcomes led to inferences about leader competency.

*I wouldn’t say I felt embarrassed for him [the CEO]*, *but I just thought*, *he’s really shown how poor his EQ is there*. [Leader Observation]

*When someone*… *is not good at humor*, *and attempts to do it*, *it becomes very awkward*. *And then it makes you question their abilities as leader in general*, *even though*, *it might not have anything to do with leadership abilities*, *just the fact that he doesn’t know when to stop*, *or he doesn’t know that it’s not funny anymore*, *it makes you question their discernment and their social skills*. [Follower Observation]

## 4 Discussion

*Comedy is an extension of intelligence*, *it’s hard to be really funny if you are not really smart*.

                                    •Will Smith

*Humor is not about comedy; it is about a fundamental cognitive function*.

                                    •Clarke [34]

The aim of this study was to explore humor use in a workplace leadership context to broadly understand the key elements that influence successful humor use. The study had three objectives. First, to identify key elements important for perceived success of humor use in a workplace leadership context. Second, to postulate how success elements underpinning humor use relate to or influence leadership effectiveness. Third, to use the findings to inform the development of a framework for effective humor use in a workplace leadership context.

This study identified the following five elements that may be critical to perceived success of humor use in a workplace leadership context: 1. reading the context, 2. intention and motivation, 3. judgment and decision, 4. skillful delivery, and 5. understanding reactions. These elements contain both cognitive and emotional processes that occur during the humor use interaction in a workplace leadership context. As reflected in the Mark Twain quotes at the beginning of the introduction, humor can impact emotions and long-lasting humor incorporates intelligence and wisdom. The link between humor and intelligence, both cognitive and emotional, is supported by research [16, 17] and was commented on considerably by participants in the study. For example, one participant stated that appropriate humor use is based on a person’s abstract reasoning ability, a component of cognitive intelligence; another participant suggested appropriate humor is about knowing when to use it and whom to use it with, a hallmark of emotional intelligence. The two quotes at the beginning of this discussion also support the implicit connection between humor and intelligence; the first quote from Will Smith, is based on his lived experience; and the other from Alastair Clarke, is based on research evidence. When both cognitive and emotional intelligence are observed in the use of humor, they are heuristically understood as a demonstration of confidence and competencies (Bitterly et al., 2017), and when exhibited in a leader, may be more likely to be perceived as evidence of effective leadership. To better understand the links between humor use and cognitive intelligence, emotional ability, as well as leadership effectiveness, established frameworks are useful.

### Cognitive intelligence, emotional intelligence and leadership effectiveness

**Fig 3:**
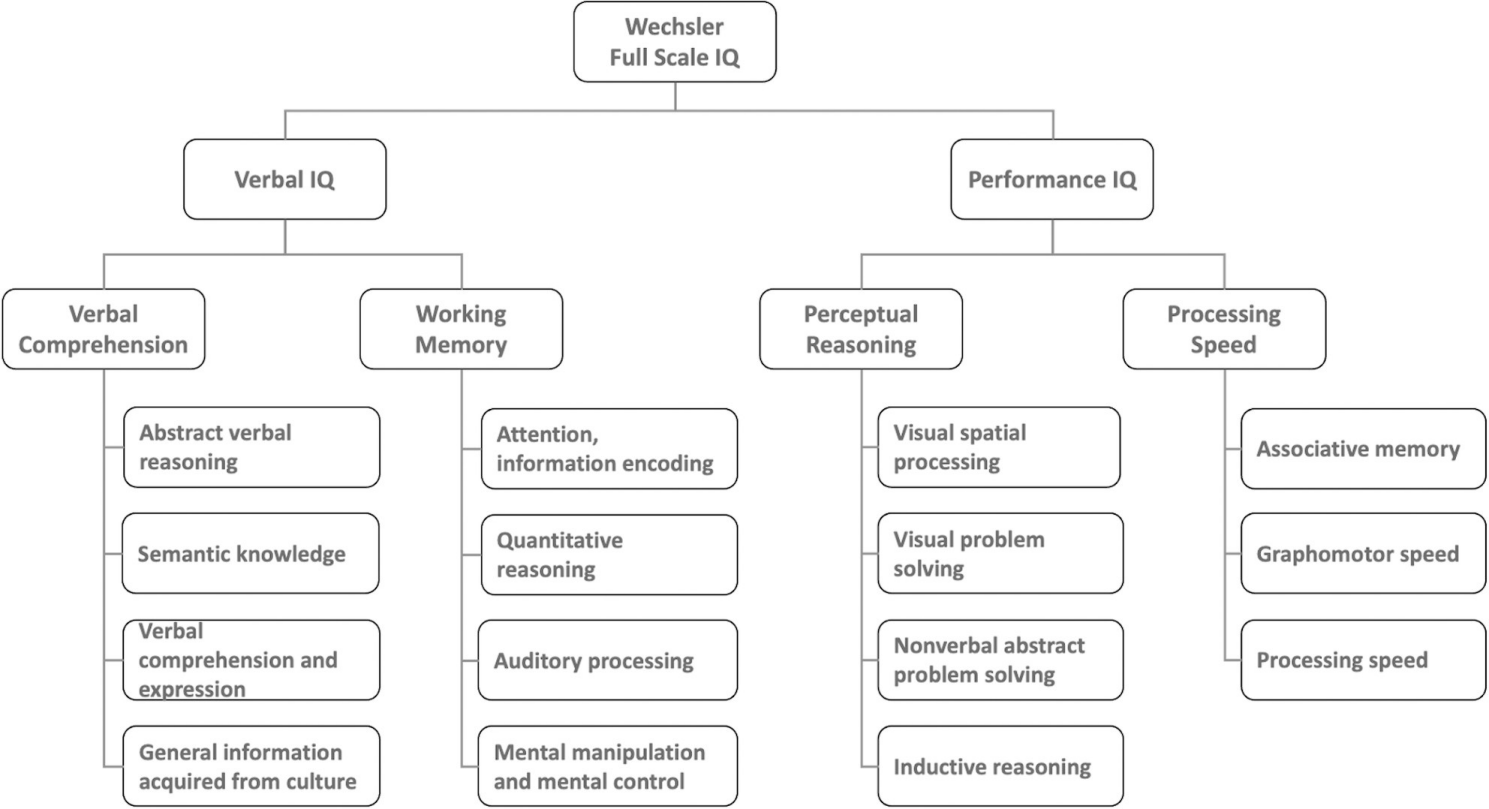
The Wechsler adult intelligence framework.

For cognitive intelligence, Wechsler’s adult intelligence framework [35, 36] can be used to understand cognitive processes and factors. The framework suggests four major components of cognitive intelligence: *verbal comprehension*, the ability to understand, learn and retain verbal information and to use language to solve novel problems; *working memory*, an individual’s ability to hold verbal information in short-term memory and to manipulate that information; *perceptual reasoning*, the ability to understand visual information and to solve novel abstract visual problems; and *processing speed*, being mental processing speed [37]. Fig 3 summarizes the four components and some proposed abilities under each component.

**Fig 4:**
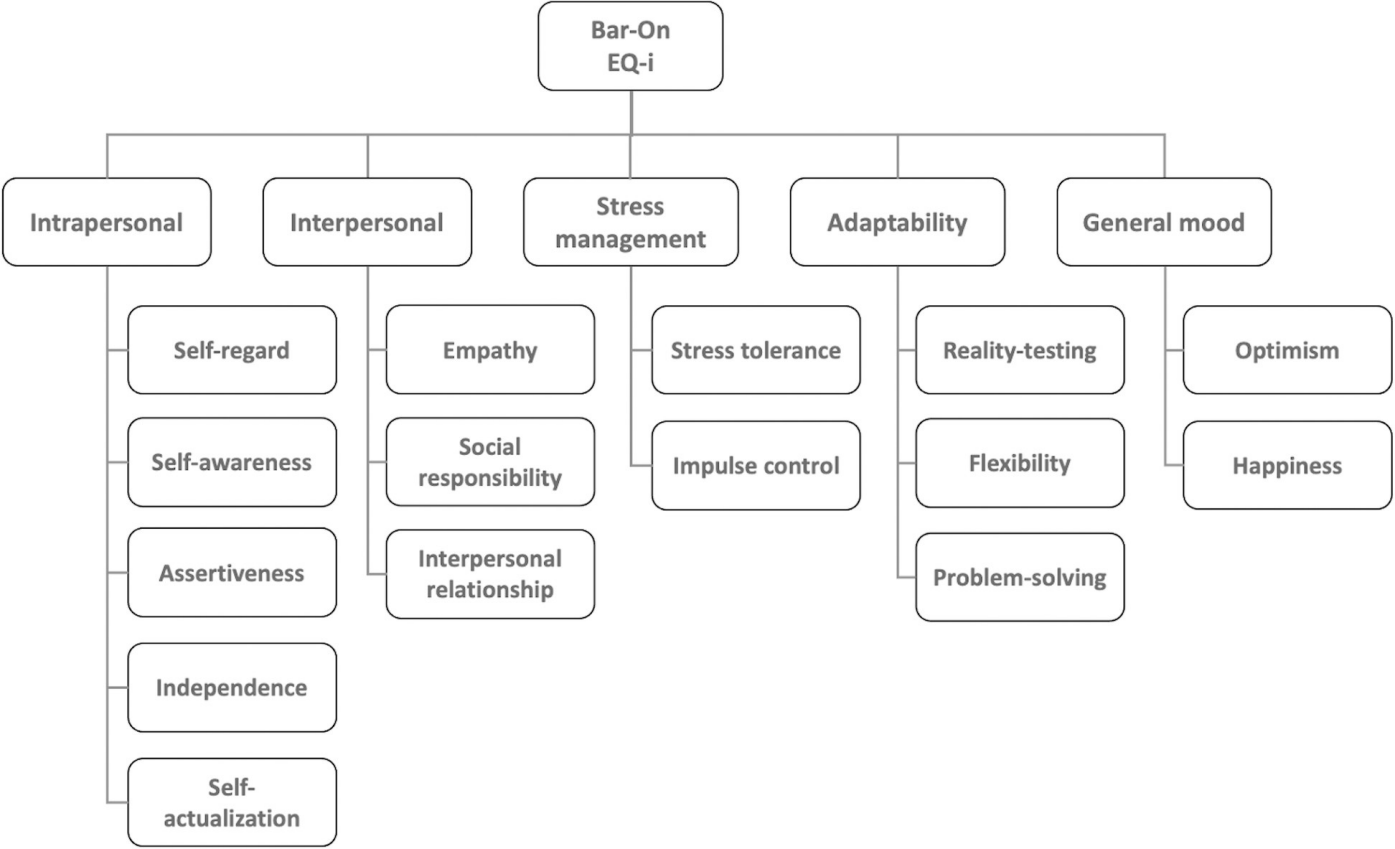
The Bar-On model of emotional-social intelligence.

Bar-On’s emotional-social intelligence model, illustrated in Fig 4, can be used to understand emotional skills and ability. The model suggests emotional-social intelligence contains five components: *intrapersonal* ability, being aware of oneself, to understand one’s strengths and weaknesses, and to express one’s feelings and thoughts constructively; *interpersonal* ability, being aware of others’ emotions, feelings, needs, to establish and maintain positive, cooperative and mutually satisfying relationships; *stress management*, the ability to effectively and constructively manage stress and control impulses; *adaptability*, effectively and flexibly manage change in the immediate situation, solving problems and making decisions; and finally, *general mood*, being sufficiently optimistic, positive and self-motivated [38].

To understand effective leadership behaviors and leadership competencies, the most commonly used framework is Avolio’s Transformational Leadership model [39]. The model proposes four basic components of effective leadership behaviors that can inspire individuals to engage in attaining organisational goals: individualized consideration, idealized influence, intellectual stimulation, and inspirational motivation. Individualized consideration refers to the leader’s ability to recognize and attend to the needs and capabilities of individual followers. Idealized influence is about being a positive role model, displaying qualities that influences others to want to become more like the leader. Intellectual stimulation refers to the leader’s ability to challenge and support followers in problem solving and decision making without criticizing. Inspirational motivation is about the leader’s ability to inspire confidence and motivation in followers through a clear vision and common goals [40]. Based on Avolio and Bass’s research, leaders that exhibit these behaviors are more effective in engaging people and attaining organisational goals.

The following section addresses the second objective of this study. It brings together the five leader humor success elements identified in this study, to postulate how leaders’ humor use can influence perceptions of leadership effectiveness and competencies through the lens of Wechsler’s adult intelligence framework, Bar-On’s emotional-social intelligence model, and the transformational leadership model.

### Success Element 1: Reading the context

Reading the context reflects an individual’s sensitivity to situational cues, nuances of the relationship dynamic, and verbal information arising from the interaction. Participants discussed this element in several ways; some focused on the people, some focused on the task, and others emphasized the environment. Reading the context encapsulates the need for leaders to be aware of the environment and being sensitive to the different demands that may exist in the situation when using humor. Components of cognitive and emotional intelligence may enable leaders to be more effective in reading the context, a process of information intake and absorption. These competencies may also enable transformational leadership behaviors.

Based on Wechsler’s adult intelligence framework, reading the context might reflect a leader’s cognitive ability in perceptual reasoning, more specifically, the ability to understand visual information components of perceptual reasoning. It could also be supported by working memory, the ability to hold verbal information in short-term memory and to manipulate that information [37]. For example, noting subtle facial expressions as indicators of humor use opportunities; or recalling and connecting relevant verbal information from past interactions to the present situation to enable witty remarks.

Through the lens of Bar-On’s emotional-social intelligence model, reading the context when using humor demonstrates the interpersonal component of emotional intelligence. It suggests the leader possesses the ability to be aware of others’ emotions, feelings and needs in a given situation; to establish and maintain positive, cooperative and mutually satisfying relationships with others [38].

One component of Avolio’s transformational leadership model is individual consideration, meaning that effective leadership considers followers’ needs and attends to them individually (Bass & Avolio, 1990). Reading the context, as a success element of humor use, is about considering the people involved in the given situation and can therefore be evidence of individual consideration in transformational leadership behavior.

If reading the context is mismanaged by a leader, observers may form an intuitive or subconscious impression that the leader is unable to pick up on situational cues and has poor interpersonal emotional intelligence. This may consequently influence the observer’s perception of their leader’s effectiveness, being that the leader is not able to recognize and attend to the needs of individual followers. This may also extend to negative perceptions of the leader’s effectiveness in other areas, for instance business acumen, where an observer may form a view that the leader may not be sensitive or attuned to business opportunities and risks in an organisational environment.

### Success Element 2: Intention and motivation

This element represents the motivation for using humor in a specific workplace situation. Some participants reported that they do not consciously formulate an intent for humor use, and that it occurs naturally in the moment. However, others reported specific reasons for using humor at work, such as easing tension or building rapport. Although this difference in participants’ reports requires further investigation, it could signify the distinction between trait and behavioral humor (expression). Trait humor refers to the likelihood of an individual experiencing or expressing a humorous state, a latent aspect of a leader’s dispositional tendency, and humor expression is a behavioral construct that indicates the frequency or the extent to which a leader expresses humor in interactions with followers.

From a cognitive intelligence perspective, the ability to formulate a clear rationale to use humor in the moment can also relate to perceptual reasoning. As discussed earlier, the first element of reading the context can be supported by the ability to understand visual information, which is the first part of perceptual reasoning. The success element of intention extends the cognitive process from understanding visual information to solving abstract problems. For example, participants reported that when they observed their audience showing signs of tiredness and agitation, they used humor to lift the energy in the room and re-engage people to the topic at hand. The cognitive process involved in observation of behavioral cues, reading the context, and having the intention to use humor may seem simple and logical retrospectively, but it requires the leader to work through three cognitive steps. First, the leader must engage their inductive reasoning skills to make sense of the subtle behaviors observed, e.g., slouching, yawning, or fidgeting, as non-verbal cues for tiredness. Second, the leader interprets the observation as a reason for intervention, e.g., tiredness may render the communication ineffective. Finally, the leader needs to engage in nonverbal abstract problem solving to formulate a strategy to intervene, such as using humor to inject energy into the room and lift audience engagement (intention), therefore resolving the issue of low engagement, and preventing the consequence of ineffective communication.

In terms of emotional intelligence, having a clear understanding of why one would use humor aligns with intrapersonal emotional intelligence. This requires self-awareness of one’s emotions and motivations, being able to formulate behavioral strategies to maintain positive self-regard and self-actualization. For example, when a leader senses frustration in the room, and perceives their authority is being challenged, they may be motivated to use humor as a strategy to ease tension or to maintain power. However, the leader needs to be self-aware of their motivation for using humor in that situation, because the outcome and reaction of followers may be different depending on whether humor use appears motivated by tempering conflict versus asserting authority. Humor can be used as a positive politeness strategy, softening the impact, and releasing the tension associated with the negative message (Holmes & Marra, 2006). However, if the leader does not have a clear awareness of their own intention when using such a strategy, and appropriately project such intention, they may be mistakenly perceived as using a power strategy, which in turn may lead to offence and unexpected reactions.

In a business sense, being able to solve abstract problems and rapidly formulate strategies is an important competency for leaders, and a crucial differentiator of successful leaders during negotiation and other situations of influence [41]. Follower positive perceptions of leader humor use intention may also ensure a positive leadership image, maintaining confidence in the workplace. This could be why positive styles of humor use, such as affiliative or self-enhancing humor, often leads to positive and desirable outcomes for both leaders and followers (Mesmer-Magnus et al., 2012). An awareness of intention and clarity of purpose when using humor, echoes the inspirational motivation component of transformational leadership. It is about having clear goals when using humor that inspire individual motivation, which improves leadership effectiveness (Bass & Avolio, 1990).

If a leader uses humor without a clear purpose or intention, it may leave followers in a confused, uncertain psychological and emotional state, and it can be taxing for followers to hypothesize the true intention of the leader’s humor use. Without this element of a clear purpose or intention when using humor, followers may perceive the leader as using humor too frequently or without cause, ultimately questioning the leader’s self-awareness, and their ability to formulate and communicate clear goals, an essential component of transformational leadership.

### Success Element 3: Judgment and decision

The third element of judgment and decision is a natural extension of the first two elements, reading the context and intention. This element describes information integration, evaluation and decision-making during the humor use process for leaders to discern if humor is appropriate for the specific context.

From a cognitive perspective, judgment and decision relies on several verbal intelligence abilities to both construct a discourse that can be perceived as funny and assess its appropriateness. While overall verbal intelligence has been found to predict humor production ability [16], some cognitive abilities included in verbal comprehension, as illustrated in Fig 3, could influence specific humor use aspects. For example, semantic knowledge has been found to be important in the creation of puns [42], and abstract reasoning can mediate the effect of sarcasm on creativity [43]. General information acquired from culture can also be critical in judging the appropriateness of humor. Some participants reported the criteria they used to determine the appropriateness of humor is based on their leadership values, which can be influenced by culture [44, 45], for example, not causing harm or forming friendships.

Working memory, may assist the leader’s ability to mentally manipulate information, evaluate the potential outcomes and reactions of humor use and to make appropriate decisions about using humor. The ability to mentally control encoded situational cues in the short-term memory, and acquired general information, such as cultural norms and values, in the long-term memory, could differentiate between leaders who can make appropriate humor use decisions from those who cannot.

A leader’s cognitive processing speed may also be essential in judgement and decision making. Some participants in this study reported that they often missed opportunities of using humor because they were not quick enough in making the appropriate judgement; others reported that they sometimes used humor inappropriately because they were not able to repress their impulse to use humor. Speed of judgement and decision-making, as well as impulse control, may therefore critically impact on the humor use outcome.

From an emotional intelligence point of view, the success element of judgement and decision can be supported by the stress management component of the Bar-On model. As illustrated in Fig 4, there are two associated competencies, stress tolerance and impulse control. As discussed in the previous element, leaders are often motivated to use humor as a discursive strategy to solve a problem in the moment and to mitigate potential negative consequences that may emerge. To maintain stable cognitive functioning required in abstract non-verbal problem solving and decision making, the leader needs to have adequate emotional stability, to tolerate the situational stress induced by the problem at hand, and the ambiguous consequence from the humor use decision. Leaders can also use humor without a clear reason or high impact consequence as previously discussed; therefore, stress tolerance may not always be required. However, even in these low stake situations, for leaders to project emotional intelligence, they should still exercise emotional impulse control–the other component of stress management, to keep excitement or irritation in check when deciding to use humor as a reaction to situational stimuli.

From a leadership effectiveness perspective, the ability to rapidly problem-solve to achieve intended objectives and do so with appropriate emotional consideration, elicits professional confidence that the followers have in their leaders (Bitterly et al., 2017). This can be perceived as evidence for intellectual stimulation—a component of transformational leadership behavior (Bass & Avolio, 1990).

If a leader cannot judge and decide when humor use is inappropriate, for example, it is used too often, or it impedes the objectives of the task at hand, it may imply a lack of cognitive and emotional control to followers and observers. This implication may further extend to the leader’s capacity to make sound business judgement and decisions.

### Success Element 4: Skillful delivery

A leader’s ability to deliver humor in a skillful manner was another theme identified in the data and involves both behavioral and verbal components. Unlike the previous elements, delivery can be directly observed, and potentially assessed. The participants recognized and reported that humor delivery is fundamentally about communication skills, including clear articulation, projecting confidence and authenticity.

The ability to successfully articulate humorous ideas indicates a high level of verbal intelligence, including semantic knowledge and the ability to express abstract social conventions and rules [36, 46]. There is also growing evidence about the gestural origin of language, sensorimotor activation in response to language processing has been demonstrated by several neurophysiological studies [47]. Therefore, both the verbal and behavioral components of humor delivery may be influenced by cognitive capacities.

The skillful delivery element may also reflect a leader’s ability to generate and maintain positive affect by being optimistic and happy, which is a component of emotional intelligence. Being able to confidently and authentically express humor might cultivate followers’ trust in their leaders, subsequently influencing leader-follower relationships in a positive way (Gkorezis & Bellou, 2016; Karakowsky et al., 2020; Kim et al., 2016). A positive and trusting relationship, in turn, promotes an open and safe workplace culture [48, 49], and an open and safe culture may further enable people to engage in positive humor events. Robert and Wilbanks [4] proposed this circular relationship between humor, emotion/affect, and workplace culture has been proposed by Robert and Wilbanks [4] as The Wheel Model of humor, theorizing that humor helps to initiate and perpetuate a cycle of individual and social-level positive emotion.

From a transformational leadership behavior perspective, this element of skillful delivery may be related to a leader’s idealized influence, which is about being a positive role model and displaying qualities that influence others to want to become more like the leader [39, 40]. The conceptual Relational Process Model of humor proposed several social and relational processes that supports the influence of humor on affect in organizations, including affect-reinforcement, similarity-attraction, self-disclosure and decreasing hierarchical salience [2].

### Success Element 5: Understanding reactions

Understanding reactions was the final element identified in the data. This element requires the leader to gauge audience reactions and respond to reactions if needed. If audience reaction to the leader’s use of humor matches the anticipated outcome, no action is required. However, if audience reaction is perceived as negative, the leader needs to judge and decide what remedial action is necessary and implement an appropriate response. This element also includes knowledge acquisition and retainment of information from the current interaction for future interactions and decision-making. Therefore, the cognitive competencies required for this element are a combination of the ones engaged by all four elements discussed previously.

Understanding reactions may also require cognitive and emotional adaptability to enable the leader to adjust appropriately when faced with unexpected reactions or outcomes. Although an unexpected outcome could be a result of mis-reading the context or unclear communication from the leader, several other situational factors (e.g. someone walking into a conversation, and overhearing things that they were not meant to hear) may also contribute to unintended reactions or outcomes and, despite the amount of care taken by the leader in using humor, the audience may not comprehend or understand the message as intended. Therefore, this element is critical regardless of how skilled a leader is in using humor. Failure to detect negative reactions from the audience or to mismanage undesirable outcomes, could signal poor leadership competency in understanding the dynamic environment and being flexible in navigating change.

### Proposed Leader Humor Success Framework

**Fig 5:**
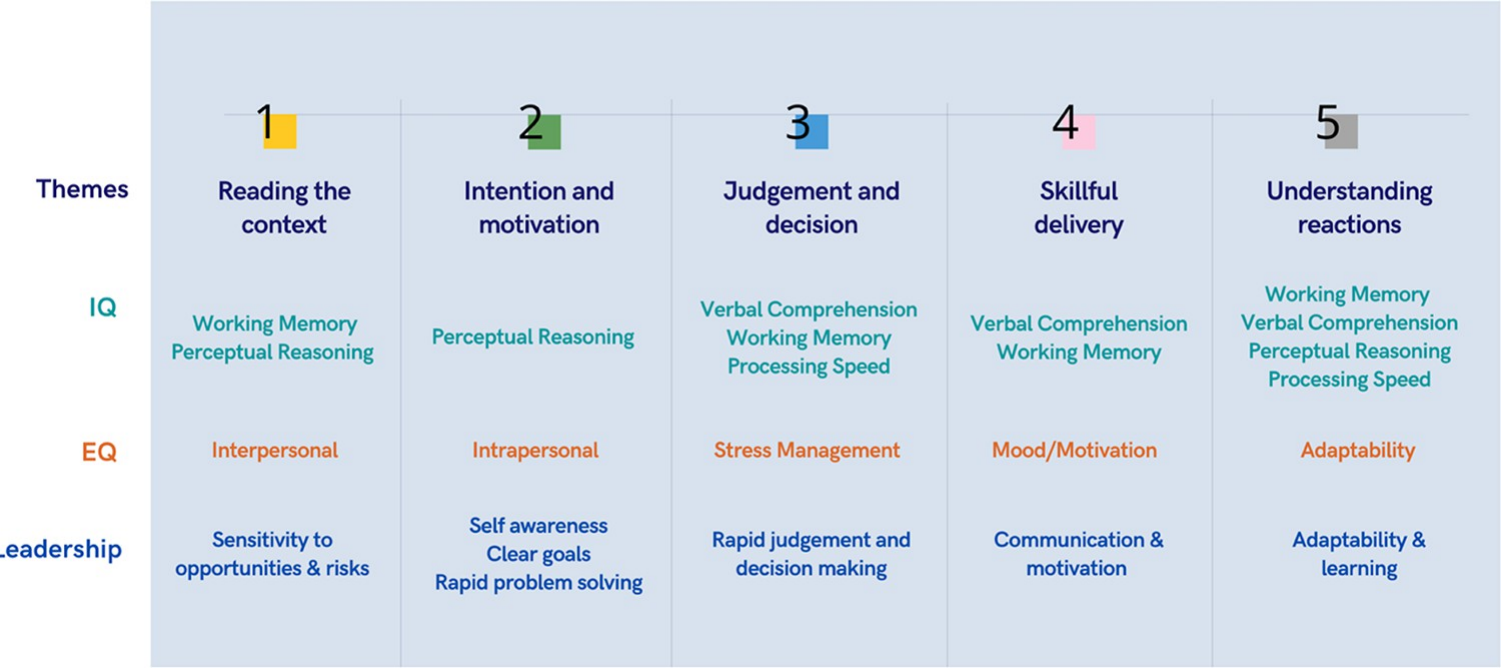
The proposed Leader Humor Success Framework.

Using the five elements identified in the data, and addressing the third objective of this study, a framework is proposed to guide the understanding of successful humor use by leaders in a workplace context, depicted in Fig 5. As can be seen in the figure, the proposed framework includes both cognitive and emotional intelligence components, as outlined in the Wechsler adult intelligence framework and Bar-On model of emotional-social intelligence.

Fig 5 illustratively summarizes the related cognitive and emotional components that may be associated with each of the five success elements. It also maps leadership competencies that can be linked or inferred by these success elements. For example, a leader’s cognitive ability in working memory, perceptual reasoning and their interpersonal emotional ability may influence leader competency in reading the context accurately when using humor. The same competency could be evident in the leader’s ability to sense opportunities and risks in a business and organisational context. The five humor success elements are hypothesized to be critical for a positive humor experience, failure or mismanagement of any element(s) could result in a perception of inappropriate use of humor in a workplace leadership context and lead to undesirable outcomes. The proposed framework is termed the Leader Humor Success Framework.

The proposed framework requires validation, and the sequence of the elements need further examination as the elements may be interchangeable or might operate as parallel processes. For example, if being humorous is a natural aspect of a leader’s personality, then the intention and motivation of using humor may always be primed. As such, the motivation element may be constantly present, meaning reading the context, and having a clear intention may appear to be absent, because they are subconscious to the leader. For leaders who are not naturally humorous, but choose to use humor as a communication tool, the humor use process would logically begin from a triggering event observed in the environment and is likely to have a more conscious process of reading the context and triggering the intent of using humor. This could also be the difference between leaders who are naturally proficient in using humor and those who are developing their humor use effectiveness. A proficient humor user might focus more on what they need to achieve and act intuitively to achieve those objectives; on the other hand, a less proficient leader may need to be more deliberate in employing the necessary humor skills needed to achieve their objective. Thus, the framework could potentially be dynamic to the leader’s level of trait humor or proficiency of humor use.

A second consideration is the element of “speed” involved in humor use. Although speed emerged in the theme of judgement and decision based on the interview data, it is possible that the effect of speed permeates the entire process of humor use. The time taken to consider each element of the humor use process may cumulatively affect the overall experience of humor spontaneity. Participants reported that leaders with spontaneous humor or quick wit are perceived as more intelligent and competent; research also supports the link between quick wit and charisma [50]. From a cognitive perspective, “processing speed” is an important factor of intelligence as defined in the Wechsler adult intelligence framework; and from an emotional perspective, speed may represent authenticity and genuineness, so that followers are less likely to question the leader’s intent, and are more likely to develop trust in a leader if the leader is perceived to use humor authentically and spontaneously.

### Significance and limitations of the study

This is the first known study to investigate the underlying cognitive and emotional competencies and skills involved in humor use in the workplace and to understand the connections between humor use and effective leadership behavior. This study also attempted to address current limitations in humor research (e.g. focusing on humor styles or the types of humor) and offer an alternative paradigm to enable researchers to investigate humor use more meaningfully. The five success elements outlined in the proposed framework, offer a new lens for understanding humor use, and may have the potential to be applied more broadly to understand effective communication in a workplace leadership context. The framework offers an insight to understand why humor may signal confidence and competence from a cognitive and emotional intelligence point of view [8].

This study does not provide the ultimate solution for mastering humor use, and the findings will need to be confirmed, but it is a first step in unveiling the processes and components that may be involved in using humor effectively. The proposed Leader Humor Success Framework also provides an alternative paradigm to Humor Styles, the Relational Process Model, and the Wheel Model of Humor, offering a foundation for researchers who are interested in understanding the underlying mechanisms and processes of humor use in addition to its effects and outcomes.

Aside from the theoretical implications, its practical application in leadership development training may also be important. The ultimate goal of investigating humor use in a workplace leadership context is to assist leaders who would like to use humor as a form of communication, thereby aiding their leadership effectiveness. The framework delineates the leadership competencies required in successfully using humor, and it maps how each of the five elements may influence the perception of transformational leadership behaviors. This allows leaders and leadership professionals to evaluate and ascertain where their strengths and development areas are in humor use, and by extension, in communication, against the five success elements. To fully realize the practical value of the framework, a validated assessment instrument needs to be constructed and developed. Researchers and scholars are encouraged to further refine the framework through scale development and validation studies.

Despite the contributions of this study to the humor literature, some limitations deserve mention. One limitation is that the uniqueness of individual leaders and their propensity to use humor naturally was not accounted for. For example, leaders who are perceived to be “naturally funny” may use humor differently to those who are not naturally funny but make a deliberate effort to use humor. Evidence show that demographic variables such as gender, age and cultural background may influence the perception and experience of humor use at work [51, 52]. The individual differences and the cultural background of the participants in this study may have influenced how they articulated and rationalized their understanding of their behavior, leadership skills, and how they used humor in a workplace leadership context. Future studies could explore individual differences in humor use. Exploring the propensity and affinity of humor use, as well as the effect that individual trait differences have on the leadership effectiveness. Other qualitative approaches could be explored to enable comparison and contrast of individual leader’s experiences based on their trait humor or proficiency of humor use [53]. Understanding both the commonalities and differences between leaders can be important to developing a sound theoretical framework.

Another limitation was that the followers’ humor use in the leadership interactions were not investigated, therefore the framework can only be generalized to people in leadership positions. Intuitively, the humor use framework should be applicable for both leaders and followers in the workplace context, however, future studies are needed to better understand the interplay and potential scope to expand the current framework.

The third limitation was that some participants had difficulty recalling critical incidents relating to humor use during the interview, even with prompting. Flanagan [28] argued that participants who reported critical incidents after a fortnight, rather than daily, could forget as many as 80% of the observed incidents. A possible implication is that several insightful incidents may not have been reported by participants in this study due to the passage of time. Future research could consider implementing ecological momentary assessments, including asking participants to journal perceived critical incidents related to humor use daily, thereby reducing reporting delays and potentially improving the quality and quantity of reporting critical incidents.

In conclusion, this study explored the underlying cognitive and emotional competencies involved in humor use by leaders in a workplace context. Through qualitative analysis of interviews and the use of critical incident technique, five key success elements of humor use were identified: reading the context, intention and motivation, judgement and decision, skillful delivery, and understanding reactions. These elements encompassed both cognitive and emotional intelligence components, highlighting the multifaceted nature of effective humor use in leadership. The proposed Leader Humor Success Framework provides a new lens for understanding and investigating humor use in a workplace leadership context. It offers a paradigm that is fit for workplace leadership and lays the foundation for the development of a new humor measurement instrument, which are the identified gaps in this area of study. Further research and application of this framework will advance the study of humor use and its pact on workplace leadership effectiveness.

## Supporting information

S1 Appendix Interview schedule. (DOCX)

S1 File Sample data. (XLSX)
