# Supplementary material for: The success elements of humor use in workplace leadership: A proposed framework with cognitive and emotional competencies
Source: PLoS One. 2024 May 31;19(5):e0304650. doi: 10.1371/journal.pone.0304650 (PMC11142654; doi:10.1371/journal.pone.0304650)
Supplement: S1 Appendix — (DOCX) [file pone.0304650.s001.docx]

## Appendix A

**Interview Schedule**

Do you identify yourself as a leader? Yes (all Questions) / No (begin with preamble to Q11)

1. Please describe your leadership context?
2. Tell me about yourself as a leader, how do you lead your team?
3. How would you describe success in leadership?
4. What do you think is important to the success of your leadership?
5. How would you describe/define humour in the workplace? (Alternate Q: What do you think about the use of humour in the workplace?)
6. Do you think humour is important to the success of leadership? Why/why not?
7. Can you describe a situation where you’ve successfully/unsuccessfully used humour in a leadership context?*

*Prompts:*

- *Context: describe the situation and what happened.*
- *Behaviours: describe your ability/lack of ability to use humour in this situation.*
- *Outcomes: what was the outcome for you/others in the situation? How did you feel about the situation?*

1. Given the opportunity, would you have done anything differently? And if so, what and why?
2. In hindsight, what do you think some of the contributing factors to the success/failure of this situation were?
3. Following the situations that you have described to me, is there anything you would like to add to the initial questions about your leadership and/or how you lead your team?

*Note: depending on which example the participant provides first (i.e. successful vs unsuccessful use) they will then be asked to provide the opposite example for questions 7-9.

Think of a leader whom you’ve worked with that regularly uses humor at work (either successfully or unsuccessfully), and bear this person in mind when answering the following questions.

1. Please describe this leader’s leadership context?
2. Tell me about this person as a leader, how do they lead their team?
3. How would you describe their leadership success?
4. What do you think is important to the success of leadership?
5. Do you think humour is important to the success of leadership? Why/why not?
6. Can you describe a situation where this leader successfully/unsuccessfully used humour in a leadership context?*

*Prompts:*

- *Context: describe the situation and what happened.*
- *Behaviours: describe the leader’s ability/lack of ability to use humour in this situation.*
- *Outcomes: what was the outcome for you/the leader/others in the situation? How did you feel about the situation?*

1. Given the opportunity, should the leader have done anything differently? And if so, what and why? (If appropriate, would you do anything differently? And if so, what and why?)
2. What do you think some of the contributing factors to the success/failure of this situation were?
3. Do you think humour is something that you’re born with or something that can be learned? Why do you think this?

*Note: depending on which example the participant provides first (i.e., successful vs unsuccessful humor use) they will then be asked to provide the opposite example for questions 16-18.
